# Sensing Organophosphorus Compounds with SWCNT Films

**DOI:** 10.3390/s21144915

**Published:** 2021-07-19

**Authors:** Mika Sahlman, Mari Lundström, Dawid Janas

**Affiliations:** 1 Hydrometallurgy and Corrosion, Department of Chemical and Metallurgical Engineering (CMET), School of Chemical Engineering, Aalto University, P.O. Box 16200, 00076 Aalto, Finland; mika.sahlman@aalto.fi (M.S.); mari.lundstrom@aalto.fi (M.L.); 2 Department of Organic Chemistry, Bioorganic Chemistry and Biotechnology, Silesian University of Technology, B. Krzywoustego 4, 44-100 Gliwice, Poland

**Keywords:** phosphines, single-walled carbon nanotubes, doping

## Abstract

Promising electrical properties of single-walled carbon nanotubes (SWCNTs) open a spectrum of applications for this material. As the SWCNT electronic characteristics respond well to the presence of various analytes, this makes them highly sensitive sensors. In this contribution, selected organophosphorus compounds were detected by studying their impact on the electronic properties of the nanocarbon network. The goal was to untangle the n-doping mechanism behind the beneficial effect of organic phosphine derivatives on the electrical conductivity of SWCNT networks. The highest sensitivity was obtained in the case of the application of 1,6-Bis(diphenylphoshpino)hexane. Consequently, free-standing SWCNT films experienced a four-fold improvement to the electrical conductivity from 272 ± 21 to 1010 ± 44 S/cm and an order of magnitude increase in the power factor. This was ascribed to the beneficial action of electron-rich phenyl moieties linked with a long alkyl chain, making the dopant interact well with SWCNTs.

## 1. Introduction

The discovery of carbon nanomaterials such as carbon nanotubes (CNTs) [1,2] and graphene [3] created new perspectives for materials science. Ever since the remarkable electrical [4,5,6], mechanical [7,8,9], thermal [10,11,12], and optical [13,14,15,16] characteristics were first observed, the research community focused efforts to apply these nanostructures in a broad spectrum of applications. Due to their favorable electrical and mechanical properties, able to offer simultaneously high strength and flexibility [17,18], they became particularly interesting components for flexible electronics or low grade heat harvesting [19]. 

However, the key problem with making these goals reality is that translation of the properties from the nano realm to the real-life scale is non-trivial. Macroscopic assemblies in the form of fibers and thin films made from carbon nanostructures experience the so-called contact resistance, which very much limits their electrical conductivity [20]. Charge transport at the boundaries of individual CNTs or graphene flakes making up the ensemble increases its resistance considerably. Furthermore, the isotropic distribution of these building blocks within the network contributes its share to the extrinsic component of resistance, which must be considered. Recent advances in the field alleviate these problems by substantially improving the internal structure alignment [21,22,23,24]. Nevertheless, the intrinsic factors of resistance remain a problem.

To overcome this issue and enhance the electrical conductivity of networks based on nanocarbon structures, one also needs to focus on the building blocks themselves. This aim can be accomplished by, e.g., improving the crystallinity of the C(sp^2^) lattice or adding dopants able to boost the charge transport characteristics. Their presence impacts the Fermi level of the material in a similar fashion, thereby improving their opportunities for commercialization by making the CNTs more conductive. 

Single-walled CNTs (SWCNTs) are amphoteric, which means that they can donate or accept electrons to become p- and n-doped, respectively [25,26]. A wide range of chemical compounds can be employed for this purpose. The first class encompasses electron-poor species such as mineral acids [27,28] or halogens [29,30]. On the other hand, the latter group typically comprises amines and their derivatives [31,32] or alkali metals [29,33]. 

An interesting category of electron-rich dopants was illustrated by Nonoguchi et al., who showed that phosphorus-bearing chemical compounds can act as powerful n-dopants with doping strength dependent on the molecular structure [34]. Addition of these species to SWCNT films considerably improved their electrical and thermoelectric properties. One of the most auspicious phosphorus dopants explored therein was 1,3-Bis(diphenylphosphino)propane. It gave enhanced conductivity from 36 to 100 S/cm and changed the Seebeck coefficient of the SWCNT network from +49 to −52 µV/K. The observed sign reversal was proof that the material was strongly n-doped. Simultaneously, pollution of the environment with phosphorous bears a number of critical consequences. It is one of the most widespread pollutants in water [35]. It affects 66% of river streams and 42% of lakes in the Unites States alone [36]. Consequently, the water quality is subject of eutrophication leading to mortality of flora and fauna [37,38].

In this work, we studied how the presence of phosphorus may be monitored by the application of electrically conducting sensors from SWCNT films. So far, similar SWCNT ensembles have been employed for sensing alcohols [39], NH_3_ [40], NO_2_ [41], and volatile organic chemicals (VOCs) [42], etc. [43]. Herein, the aim was to resolve the critical structural features of an organophosphorus compound to make the detection by the SWCNTs the strongest. A selection of model organic phosphine derivatives was employed: 1,3-Bis(dimethylphosphino)propane (linear alkyl substitution on P atom), 1,3-Bis(dicyclohexylphosphino)propane (cyclic alkyl substituent on P atom), and 1,3-Bis(diphenylphosphino)propane (aryl substituent on P atom). The impact of length of the alkylene group was studied using 1,6-Bis(diphenylphosphino)hexane. The change of microstructure/purity of the SWCNT films upon doping was analyzed. Then, electrical conductivity, Seebeck coefficients, and power factors were established. Finally, modeling the electronic density distribution of the dopants enabled us to hypothesize the structure of an organophosphorus most sensitively detected by SWCNTs. 

## 2. Materials and Methods

### 2.1. Compounds and Materials

Large-diameter SWCNTs (Tuball™; OCSiAl, Leudelange, Luxembourg) were evaluated in the form of ensembles. The following organophosphorus dopants were used: 1,3-Bis(dimethylphosphino)propane (dmpp), 1,3-Bis(dicyclohexylphosphino)propane (dcpp), 1,3-Bis(diphenylphosphino)propane (dpp), 1,6-Bis(diphenylphosphino)hexane (dpph). All of them were procured from Sigma-Aldrich (St. Louis, MO, USA). Acetone and toluene engaged as a medium for preparing SWCNT networks were obtained from Avantor, Gliwice, Poland. Ag conductive paint (SCP03B; Electrolube, Ashby-de-la-Zouch, UK) was employed to prepare specimens for characterization of their electrical conductivity.

### 2.2. Manufacture of Free-Standing SWCNT-Based Films

**Figure 1:**
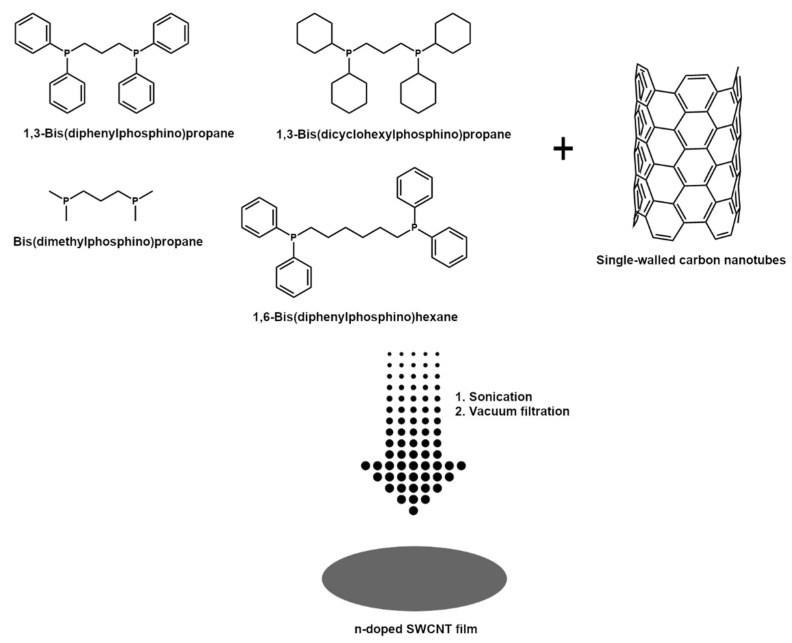
Manufacture of n-doped SWCNT films by vacuum filtration in the presence of organophosphorus compounds.

SWCNT films were manufactured by a technique developed in-house [44]. The difference between the reference and the newly reported approach is that a different class of doping agents was used herein to elucidate the mechanism of their action (Figure 1). 

Briefly, 150 mg of SWCNTs kept in a desiccator were added to 80 mL of acetone and toluene mixture (1:1 by weight). Then, an appropriate amount of the above-mentioned doping agents was introduced to establish a 0.1 M concentration in this medium (for sensitivity experiments the concentration was varied from 0.001 M to 1 M for dpph). 

Afterward, the mixture was homogenized by ultrasonication at 100% amplitude (UP200St sonicator; Hielscher, Teltow, Germany) for 10 min over an ice bath. Such an amount of time was sufficient to reach a uniform dispersion. It was filtered under reduced pressure using PTFE membrane filters (pore size: 0.45 µm, diameter: 47 mm; Fisherbrand, Ottawa, ON, Canada). Due to the low adhesion of SWCNT films to PTFE, they were easily peeled off the surface. Dopant-free SWCNT films were also made as a reference. 

### 2.3. Characterization

Raman spectroscopy (inVia Renishaw system, Wotton-under-Edge, UK) was used to gauge the structural perfection of SWCNTs and the impact of doping on the electronic characteristics. The spectra were acquired at the excitation wavelength of λ = 633 nm from 100 to 3000 cm^−1^. Laser power was kept to the minimum (0.01% total power) to ensure that the sample was not heated by absorption of radiation [32]. Mean values of I_D_/I_G_ ratios with established standard deviations are reported along with the position of the G^+^ component to study the doping effect. Multiple acquisitions at different locations of the samples eliminated the possible impact of sample inhomogeneity and background noise, respectively.

Scanning electron microscope (SEM, JEOL JSM-7500FA, Tokyo, Japan) visualized the microstructure of the SWCNT films before and after doping. The experiments were conducted at the acceleration voltage of 15 kV. The material was well conductive, so it was not sputtered with metal for imaging.

The electrical conductivity of the SWCNT films was gauged in a four-terminal configuration. The specimens (3 mm × 40 mm) were cut out from the SWCNT films obtained after filtration. Then, they were attached to custom-designed sample holders. The terminals were made of Cu to give current-carrying and voltage-sensing pairs. To ensure no issues with electrical and mechanical contact between SWCNTs and Cu, Ag conductive paint (SCP03B; Electrolube, Ashby-de-la-Zouch, UK) was applied at the interface. A source meter (Keithley 2450 SourceMeter, Cleveland, OH, USA) measured the conductivity in this setup. Conductance was recalculated to conductivity by taking into account the samples’ dimensions. The thickness was measured with a micrometer screw gauge (Electronic Universal IP54, Linear Tools, Dunstable, UK).

Seebeck coefficients of the materials were obtained using a custom-made apparatus (SeebCam, LBR, Lublin, Poland) across the 30–100 °C temperature range. The samples were mounted on a board enclosed in a sealed chamber free of air to minimize the effect of convection. The ends of the samples were then put in contact with resistive heaters and temperature sensors, which established a temperature gradient and monitored the temperature, respectively. The difference in electric potential was measured at the temperature gradient of 5 °C. The reported values were averaged across the indicated temperature range. In all the cases, multiple measurements were conducted to ensure the statistical significance of the obtained data. 

Molecular models of the dopants were drawn by Avogadro: an open-source molecular builder and visualization tool [45]. The structures were optimized in Universal Force Field (UFF). Gaussian 09W (B3LYP/6-31G(d) model) was used to approximate the atomic charges and thus the electron densities of the molecules under investigation in Avogadro.

## 3. Results

**Figure 2:**
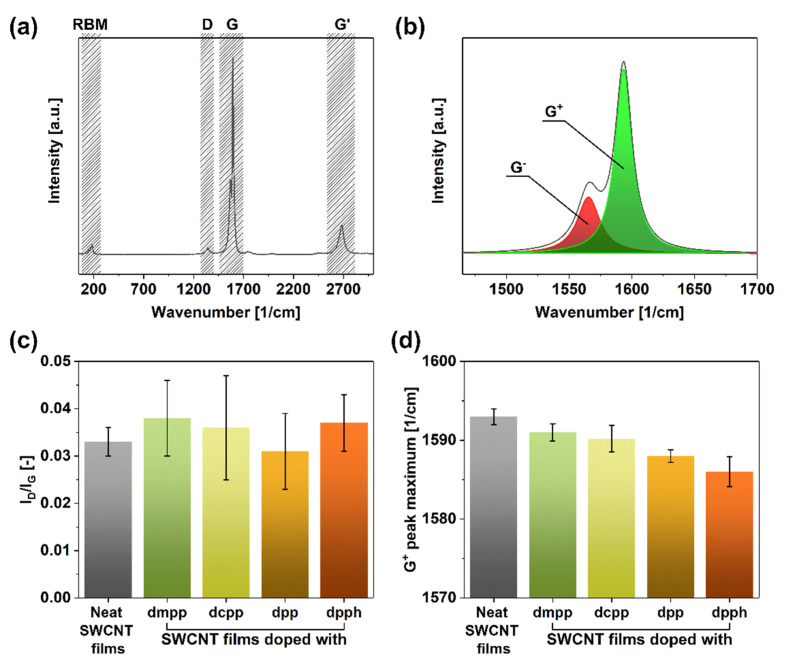
(**a**) Full Raman spectrum of a neat SWCNT film, (**b**) magnification of the G peak area with exemplary deconvolution into G− and G+ components, (**c**) I_D_/I_G_ ratios of SWCNT films before and after doping, and (**d**) corresponding recorded positions of G+ peak maxima after deconvolution.

We began the analysis by investigating the crystallinity of evaluated SWCNT networks by Raman spectroscopy (Figure 2). The SWCNT present in the parent unmodified material (Figure 2a) manifested typical features such as the radial breathing mode (RBM) (indicative of single- or double-walled character of the sample) as well as D (corresponding to sp^3^ carbon atoms), G (stemming from the presence of sp^2^ carbon atoms), and G’ bands. What is more, a clear split of the G band into G− and G+ components was observed (Figure 2b), which confirmed that the material was of single-walled character [46]. 

One of the most straightforward techniques of estimating the purity of SWCNTs is to establish the I_D_/I_G_ ratio, which quantifies the relative amount of impurities (sp^3^ carbon atoms from SWCNT defects or non-SWCNT carbon to D feature intensity) to pristine SWCNT material (sp^2^ carbon atoms to G feature intensity) [46]. Firstly, the results obtained in this study showed that the starting material was pristine as the I_D_/I_G_ ratio was as low as 0.033 ± 0.003 (Figure 2c). Secondly, upon introducing the dopants, neither of the samples exhibited a statistically significant increase in the D-band intensity. Instead, the values of the I_D_/I_G_ ratios stayed within the 0.031–0.038 range, so the dopant-SWCNT interactions are physical rather than chemical. The slight discrepancy can be assigned to the measurement error since the intensity of the D-peak was very low. 

More insight regarding the action of the dopants on the SWCNTs can be obtained from the analysis of the position of the G+ peak maxima [47]. According to the literature, when a red-shift of this feature occurs, it indicates an upwards shift to the Fermi level caused by n-doping. While the G+ peak maximum was at 1593 cm^−1^ for the untreated material, once organophosphorus dopants were introduced to the network, a clear red-shift was observed (Figure 2d). The addition of dmpp, dcpp, dpp, and dpph moved the G+ peak maximum to 1591, 1590, 1588, and 1586 cm^−1^, respectively. Based on these measurements, dpph appeared as the strongest dopant, as it repositioned this feature by as much as 7 cm^−1^. This is noteworthy as SWCNTs are naturally p-doped by oxygen in the ambient [48], so the specific dopant had to be powerful enough to overcome this effect. 

**Figure 3:**
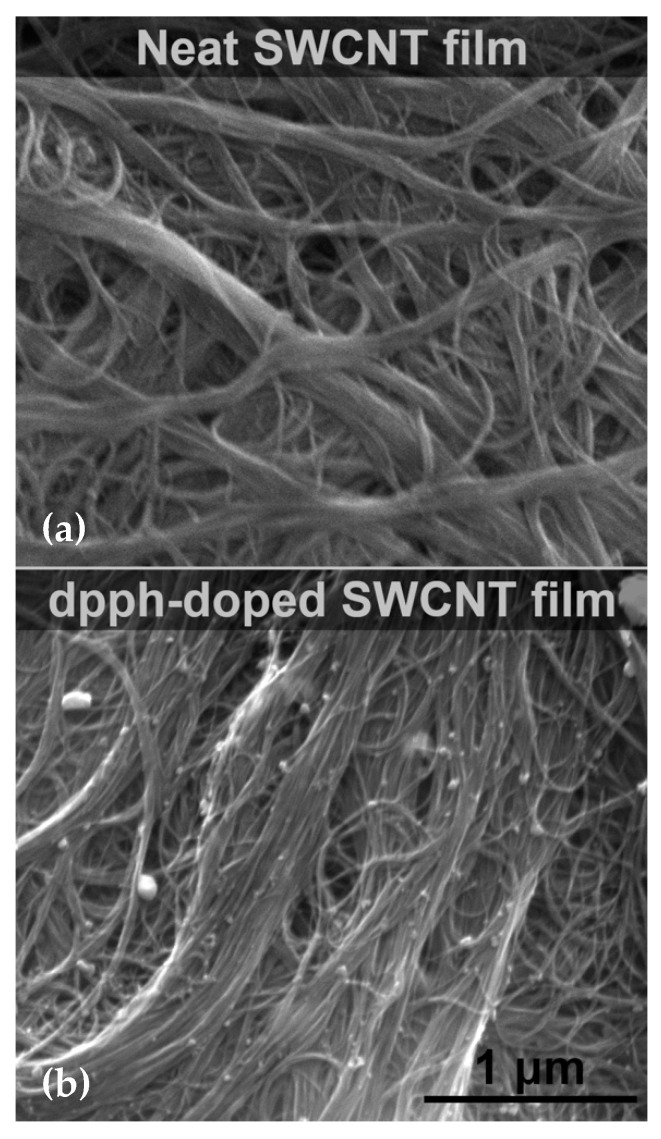
SEM micrographs of (**a**) neat SWCNT film and (**b**) upon doping with dpph.

Furthermore, SEM imaging was conducted to probe for possible changes to the microstructure of the material upon dopant addition (Figure 3). The neat SWCNT film (Figure 3a) showed isotropic structure as anticipated for buckypapers made by filtration. The material was arranged into bundles of a considerable number of SWCNTs. The employed SWCNTs had an average diameter of 1.6 nm, while the diameter of the bundles reached up to hundreds of nanometers. No impurities could be discerned in the images. 

Upon doping the SWCNT film with dpph, which showed the most substantial shift to the G+ peak maximum, there was no obvious change to the alignment or porosity of the ensemble (Figure 3b). The only difference was the presence of dpph molecules, which are solid at room temperature, so they were visualized on the surface. It should be noted that the dopant particles were well dispersed throughout the structure of the material, which can already partially explain why the observed doping was so effective (*vide infra*). 

**Figure 4:**
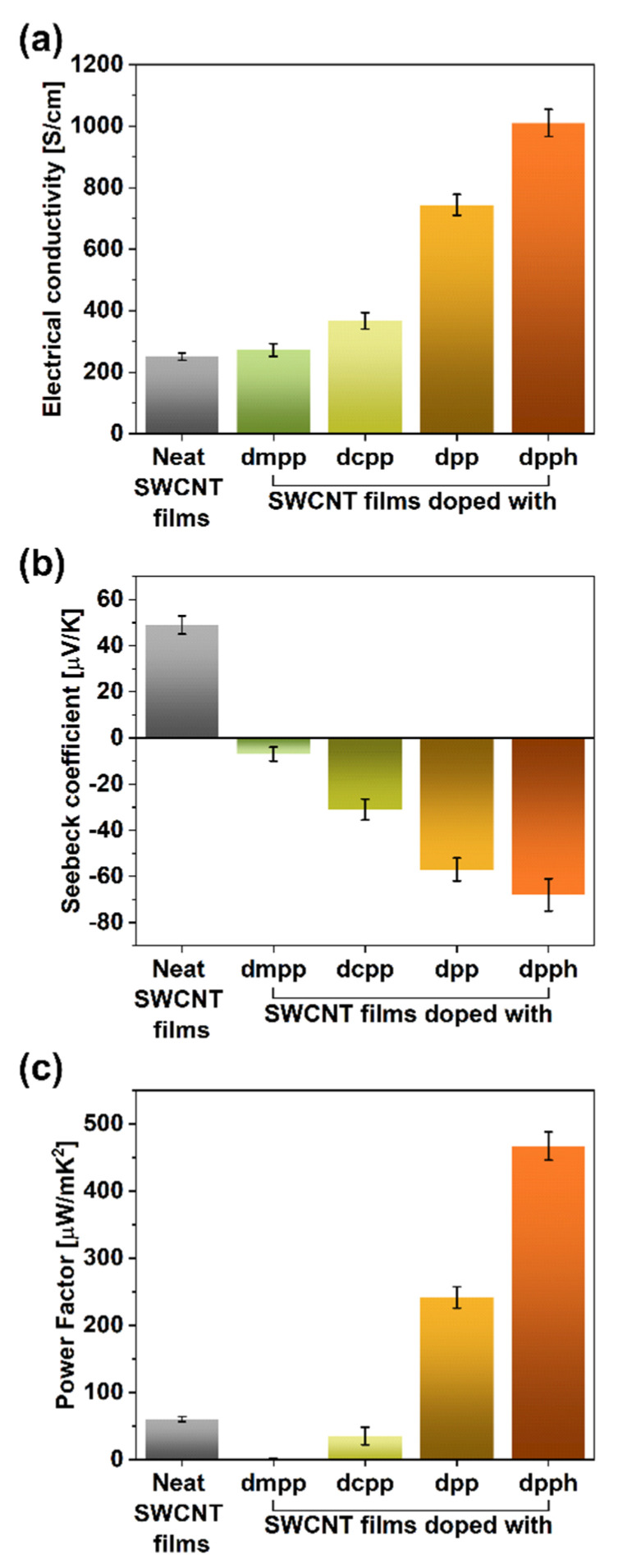
(**a**) Electrical conductivity values, (**b**) Seebeck coefficients, and (**c**) power factors of the SWCNT films before and after doping with organophosphorus compounds.

The electrical and thermoelectric properties of the SWCNT films were measured to study this effect in more detail (Figure 4). The electrical conductivity of the parent material was at the level of 251 ± 12 S/cm, which matches the values reported in the literature [19] (Figure 4a). The introduction of the organophosphorus dopants clearly enhanced this parameter. While the addition of dmpp or dcpp caused the electrical conductivity to increase to 272 ± 21 and 367 ± 27 S/cm, much more tangible benefits brought the introduction of dpp or dpph. In these cases, the electrical conductivity was enhanced to 744 ± 34 and 1010 ± 44 S/cm, respectively. Therefore, the addition of dpph at the point of SWCNT film formation managed to cause a four-fold boost to this property. The extent to which the electrical conductivity was increased paralleled the magnitude of the G+ peak shift. 

The measurement of Seebeck coefficients showed that once the organophosphorus compounds were introduced, the charge transport within the material became dominated by electrons (displayed by the sign change of the Seebeck coefficients; Figure 4b) following previous findings [34]. Notably, the addition of dpph changed the Seebeck coefficient from +49 (starting SWCNTs) to −68 μV/K (doped SWCNTs), which once again proved a strong influence of these doping species in particular. 

A measure that can gauge a material’s suitability to act as a thermogenerator is the so-called power factor (PF). It considers the electrical conductivity (σ) and Seebeck coefficient (α) while neglecting the impact of thermal conductivity. It is quantified according to the following formula: PF = α^2^∙σ. The PF values for all the evaluated dopants are established in Figure 4c.

Colossal changes to the capability of the material to generate thermopower were witnessed. The PF value of the parent material, which was 60.27 μW/m∙K^2^, decreased to a mere 1.44 μW/m∙K^2^ for the sample doped with dmpp. On the other hand, once dpph was added, one order of magnitude increase was observed. As a result, the PF value of 467 μW/m∙K^2^ was recorded for such formulation, which is among the highest thermoelectric performance reported so far [19]. This encouraging improvement was caused by the synergistic action of electrical conductivity and Seebeck coefficient, both of which increased considerably after the treatment. Analogously, the deterioration of the thermoelectric capabilities for SWCNT samples doped with dmpp and dcpp resulted from the decrease of Seebeck coefficients, which have a quadratic impact on the PF values. 

**Figure 5:**
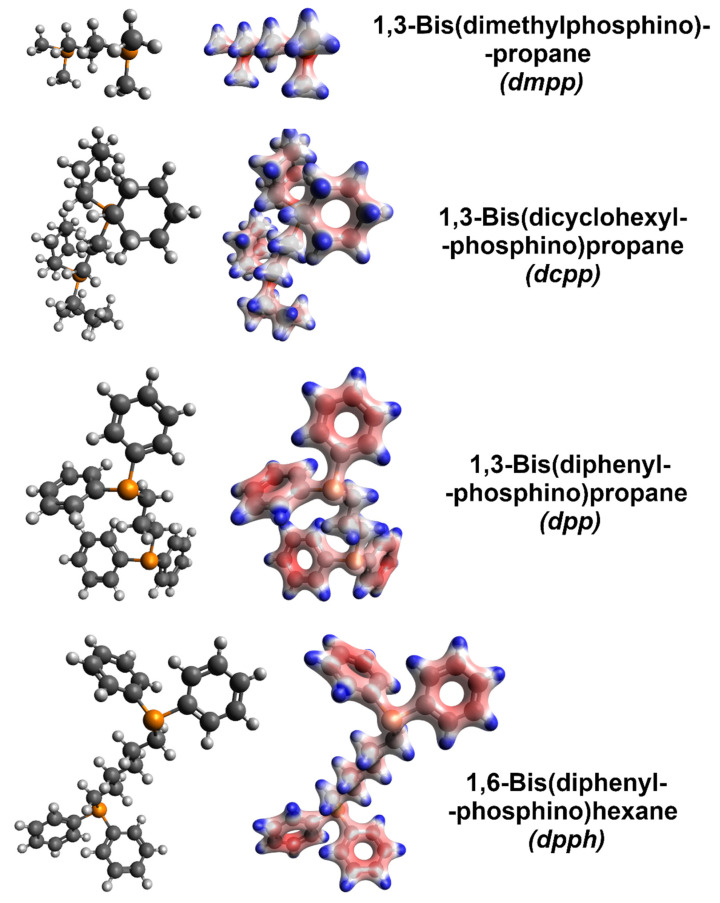
Left column: bare molecular models of the organophosphorus dopants (grey—carbon, white—hydrogen, and orange—phosphorus). Right column: corresponding molecular models with electron densities overlaid obtained by computation (red—negative charge and blue—positive charge).

Molecular models with electron densities were calculated (Figure 5) to decipher the radically different influence of various organophosphorus compounds explored in this work). Firstly, the addition of dmpp caused only slight improvement to the electrical conductivity, while the Seebeck coefficient was very much reduced. Among the explored dopants, this was the only aliphatic and acyclic member, which suggests that such characteristics cannot provide the proper dopant-SWCNT interaction necessary to enhance the electrical and thermoelectric properties of the material. Furthermore, according to the calculated electron densities, the negative charge is localized at the phosphorus atoms, which is not optimal (vide infra). On the other hand, dcpp, also an aliphatic compound, could generate a negative charge away from phosphorus atoms. This modification already resulted in moderate improvement to the electrical conductivity and made the Seebeck coefficient of SWCNTs more negative. However, a substantial boost to these parameters was obtained only when the organophosphorus dopants (dpp and dpph) were equipped with phenyl groups, where the negative charge was concentrated. Such functional groups can interact well with the surface of SWCNTs by van der Waals forces (π–π interactions, in particular). With this in mind, the charge needs to be effectively donated to the SWCNTs for the doping to be potent. Lastly, the performance of dpph was substantially better than that of dpp. We hypothesize that the six methylene groups in dpph give more flexibility to the dopant. Consequently, such doping species can assume a conformation promoting the interactions between the negatively charged phenyl groups and the SWCNT side-wall. The difference in electron density distribution between dpp and dpph may be disregarded from consideration. For both the dopants, the amount of negative charge in the phenyl substituents is indistinguishable. 

**Figure 6:**
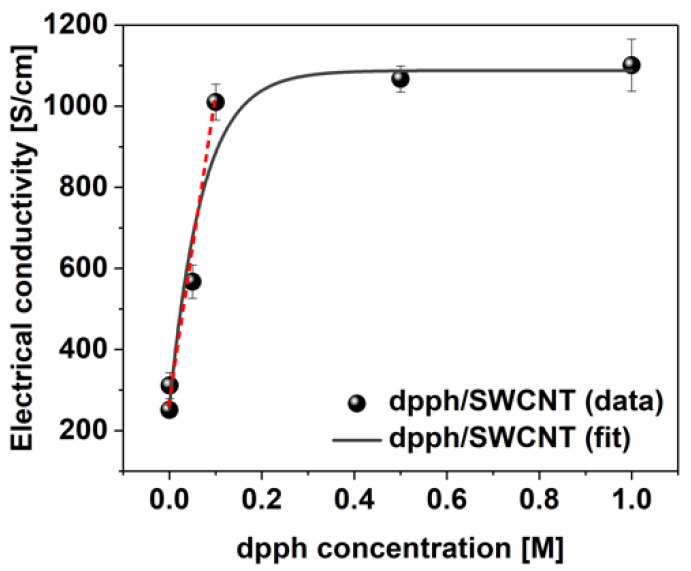
Electrical conductivity values of the SWCNT films before and after doping with dpph as a function of dopant concentration.

Lastly, the sensitivity of SWCNT films towards detection of organophosphorus compounds was evaluated using dpph across a 0.001 M–1 M concentration range (Figure 6). Tangible change in electrical conductivity from 251 ± 12 to 311 ± 32 S/cm can already be detected at the lowest dopant concentration of 0.001 M. As the concentration of dpph is elevated, a linear increase in conductivity is evident up to 0.1 M concentration. Above this point, only negligible enhancement of electrical conductivity is observed indicating that the predominant number of the sites available for dopant adsorption, which could impact the Fermi level of the SWCNTs, are already occupied. 

## 4. Conclusions

In summary, we illustrated how the presence of organophosphorus compounds may be sensed by monitoring the electrical and thermoelectric properties of ensembles from SWCNTs. Four model dopants were evaluated—1,3-Bis(dimethylphosphino)propane (dmpp), 1,3-Bis(dicyclohexylphosphino)propane (dcpp), 1,3-Bis(diphenylphosphino)propane (dpp), and 1,6-Bis(diphenylphosphino)hexane (dpph) to unravel the electronic and structural parameters of an ideal chemical compound for this purpose. The results showed that dpph organophosphorus dopant improves the electrical conductivity four-fold and notably boosts the Seebeck coefficient of the material by changing its sign and increasing its absolute value. As a consequence, the power factor of the material was augmented by order of magnitude, i.e., from 60.27 to 467 μW/m∙K^2^. 

The crucial attribute of an effective organophosphorus dopant in this study turned out to be the presence of phenyl groups connected directly with the phosphorus atoms to donate the charge to SWCNTs in a facile manner. Simultaneously, the importance of a sufficiently long alkyl chain was demonstrated as the dopant needs to assume an appropriate conformation to promote the charge transfer. 

This study showed that to tune the electrical and thermoelectric properties of SWCNTs effectively, the know-how of organic chemistry is indispensable. In light of the preceding, further molecular design may pave the way to the more effective exploitation of the opportunities provided by nanocarbon materials. Simultaneously, the progress gained in this area would also reveal how to sense phosphorous pollution with such materials in the most selective and sensitive fashion. 

## Data Availability

Data regarding this article is available from the corresponding author upon a reasonable request.
